# Knowledge, Attitudes, Practices (KAP) of Italian Occupational Physicians towards Tick Borne Encephalitis

**DOI:** 10.3390/tropicalmed5030117

**Published:** 2020-07-16

**Authors:** Matteo Riccò, Giovanni Gualerzi, Silvia Ranzieri, Pietro Ferraro, Nicola Luigi Bragazzi

**Affiliations:** 1 AUSL–IRCCS di Reggio Emilia, Occupational Health and Safety Service on the Workplaces of the Local Health Unit of Reggio Emilia (SPSAL), Via Amendola n.2, I-42122 Reggio Emilia, Italy; 2 Department of Medicine and Surgery, School of Medicine, University of Parma, Via Gramsci n.14, 43123 Parma, Italy; gualerzi@gmail.com; 3 Department of Medicine and Surgery, School of Occupational Medicine, University of Parma, Via Gramsci n.14, I-43123 Parma, Italy; silvia.ranzieri@studenti.unipr.it; 4 ASL Foggia, Department of Prevention, Occupational Health and Safety Service on the Workplaces of the Local Health Unit of Foggia, Piazza Pavoncelli 11, I-41121 Foggia, Italy; dott.pietro.ferraro@gmail.com; 5 Laboratory for Industrial and Applied Mathematics (LIAM), Department of Mathematics and Statistics, York University, 4700 Keele Street, Toronto, ON M3J 1P3, Canada; bragazzi@yorku.ca

**Keywords:** *Ixodes ricinus*, knowledge, risk perception, tick, tick-borne encephalitis (TBE), occupational physicians

## Abstract

Tick-Borne Encephalitis (TBE) is an occupational health threat with increasing incidence in the geographic area of Italy. Despite this, TBE vaccination rates have ranged from 10% to 40% in Italy, even in at-risk workers. The reasons for this low rate are investigated in this present study of the knowledge, attitudes, and practices of occupational physicians (OP) regarding TBE disease and vaccination in at-risk workers. A total of 229 OP participated in an internet-based survey by completing a structured questionnaire. Adequate general knowledge of TBE disease was found in 58% of OP. Accurate perception of TBE risk in occupational settings was found in 20%. TBE vaccination for at-risk workers was recommended by 19%. Willingness to recommend TBE vaccination was more likely by OP practicing in endemic areas (Odds Ratio 3.10, 95% confidence intervals 1.47–6.55), who knew the existence of the term “arboviruses” (3.10, 1.29–7.44), or exhibited a better understanding of TBE (2.38, 1.11–5.12)—and were positive predictors for promoting TBE vaccine, while acknowledging that TBE as a severe disease was a negative one. Tick-borne disorders in Italy are a still rare (but increasing) occupational health threat, and vaccination gaps for TBE virus may find an explanation in OP incomplete knowledge of evidence-based recommendations.

## 1. Introduction

Tick-borne encephalitis (TBE) is a potentially lethal vaccine-preventable infection of the central nervous system (CNS) caused by an arbovirus (TBE virus, or TBEV) included within the Flavivirus genus, Flaviviridae family [1]. Overall incidence rates for TBE in most Western European countries have ranged between 0.4 and 0.6/100,000 over the years 2011–2015; but notification rates in certain areas (e.g., Czech Republic, Estonia, Lithuania, Slovenia) have largely exceeded 5 cases/100,000 population/years [2,3,4,5]. With mortality rates up to 2% and long-term neurological complications in around 10% of patients, TBE has emerged as an increasing cause of morbidity and mortality [1,2,3,6,7,8,9]. The geographical distribution of the competent vector, *Ixodes* spp., is expanding as an effect of climate change. For this reason, previously spared countries have become endemic for TBE [8,10,11], particularly in northern regions and in mountainous territory, whose forests support the life cycle of *Ixodes ricinus* [1,2,3,8,9].

For instance, Italy has historically been considered as a relatively low-risk region, having no cases reported until 1978, and a total of 456 cases notified between 2000 and 2016 [2,3,7]. With 0.1 incident cases/100,000 population/year and no TBE-related deaths since 2016, Italy remains, actually, a low-incidence country [4]. Since 2015, a total of 135 cases have been reported; the majority of them clustered in North-Eastern Subalpine Regions of Trentino-South Tyrol, Friuli-Venezia-Giulia, and Veneto [3,4,12]. Recent estimates suggest that some smaller areas in the aforementioned regions of Trentino (1.0 case/100,000 inhabitants for the province of Trento as a whole during 2000–2013, peaking to 41.6 in the municipality of Tres), Friuli-Venezia-Giulia (1.0 case/100,000 inhabitants for the whole province of Udine, but 181.2/100,000 for the community of Tramonti di Sopra in the time period 2000–2013) [3,12], and Veneto (e.g., 5.95/100,000 population in the province of Belluno 2007–2018, peaking to 35.9/100,000 in the municipality of Limana in 2000–2013), [2] largely exceed national TBE estimates [3,8,9,12,13], and could be classified as highly endemic areas (i.e., >5 cases/100,000 inhabitants/year) according to current World Health Organization (WHO) definition [1,2,3,6,7,8,9,12].

As a consequence, TBE has become a significant health threat for outdoor workers in North Eastern Italy [6,7,14,15,16], and the Italian Ministry of Health has recommended TBE vaccination for forestry workers, farmers, and veterinarians from endemic areas [1,2,3,8,12]. Even though official data on TBE vaccination rates in Italy are lacking, some reports hint towards figures ranging between 10% and 40%, even in high-risk groups from highly endemic areas [2,3,4,7,12]. Such recommendations have been issued by means of the National Immunization Prevention Plan 2017–2019 (Piano Nazionale Prevenzione Vaccinale—PNPV), a guidance document for immunization policies [17,18]. Application of immunization policies for workplaces is a main issue for occupational physicians, the medical professionals responsible for health promotion in workplaces [18,19,20,21]. More precisely, Italian occupational physicians are not only requested to properly tailor national recommendations to specific occupational settings, but they should recall the vaccination status of the assisted workers, informing them about the pros and cons of recommended vaccinations [18,20,21]. Moreover, occupational physicians (OP) should actively inform workers about occupational risk factors, including vectors and microorganisms. As prevention of TBE may also benefit from several environmental (e.g., pesticide treatment of the working environment) and behavioral (e.g., promoting self-assessment for tick bites at the end of outdoor activities) interventions, the assessment of knowledge (i.e., the awareness of official recommendations), attitudes (i.e., propensity towards a certain intervention), and practices (i.e., actual application of such intervention)—or knowledge, attitudes, practices (KAP)—of occupational physicians towards TBE and TBE vaccine, they can be useful in order to improve the health and safety of high-risk outdoor workers [10,14,22].

Although studies about outdoor workers have assessed their KAP toward TBE preventive measures [10,14,23,24,25,26], occupational physicians have been scarcely investigated. Therefore, the main endpoint of this study is to assess KAP of occupational physicians concerning TBE preventive measures, particularly TBE vaccination, in order to assess factors associated with TBE vaccine acceptancy, and alternative or complementary preventive practices.

## 2. Materials and Methods

Study design. A cross-sectional questionnaire-based study was performed between 01/02/2020 and 28/02/2020, involving occupational physicians participating in seven different private Facebook group pages and four closed forums, focusing on occupational medicine, and whose applications were officially limited to occupational physicians. The group pages had approximately 2034 unique members, but no information could be obtained regarding cross-inscriptions, not even how many of these members were actively using Facebook.

To post the study invitation, the chief researcher contacted the administrators, requesting preventive authorization to post the link to the questionnaire, including a short description of the aims of the survey. Facebook users who clicked on the invitation texts were provided with the full study information, an opportunity to give their informed consent, and a web link to the survey (Google Forms; Google LLC; Menlo Park, CA, USA). The survey was conducted in Italian. To be included in the sample, the participant was supposed to be living and working in Italy as an occupational physician. If a potential participant was found not to match the inclusion criteria, the survey closed down. The survey was anonymous, and no personal data, such as name, IP address, email address, or personal information unnecessary to the survey, was requested, saved, or tracked. No monetary or other compensation was offered to the participants.

Questionnaire. The questionnaire was formulated in Italian, and its test–retest reliability was preventively assessed through a survey on 10 occupational physicians completing the questionnaire at two different points in time. The testing questionnaires were ultimately excluded from the final analyses. All questions were self-reported, and not externally validated. An English translation of the questionnaire is available on request from the corresponding author. The final questionnaire included the following sections:

1. individual characteristics: age (by decennial groups), sex, whether they had encountered at least a TBE case in their practice (yes vs. no), and the Italian Region where the professional mainly worked and lived. The latter factor was eventually dichotomized as endemic vs. non-endemic for TBE;
2. knowledge test: participants were initially requested whether they knew the meaning of the term “arbovirus”, being able to explain it. Participants then received a knowledge test containing a set of 20 true–false statements, elaborated through extensive literature review, covering typical misconceptions about arboviruses (e.g., “TBE vaccine is effective also against Lyme disease”; FALSE) [5,9,23,24,25,26,27,28,29,30]. Across the knowledge test, TBE was always reported as “TBE” or “tick-borne encephalitis” (in English), avoiding the Italian translation (i.e., “Encefalite da zecca”). A General Knowledge Score (GKS) was then calculated as the sum of correctly and incorrectly marked recommendations: when the participants answered correctly, +1 was added to a sum score, whereas a wrong indication or a missing/“don’t know” answer added 0 to the sum score. GKS was then dichotomized by median value in higher vs. lower knowledge status;
3. risk perception: participants were initially asked to rate the perceived severity (C^INF^) and the perceived frequency (I^INF^) of TBE in agricultural and forestry settings by means of a fully labeled 5-points Likert scale. The available options ranged from “not significant” (i.e., “of no significant concern in daily practice”, score 1) to “very significant” (i.e., “of very high concern in daily practice”, score 5). As perceived risk has been defined as a function of the perceived probability of an event and its expected consequences [21,27], a Risk Perception Score (RPS) was eventually calculated as follows:
  RPS = I^INF^ × C^INF^ (1)
4. attitudes and practices: we inquired participants whether they recommend TBE vaccine for high-risk groups (yes vs. no). A series of possible interventions for prevention of arboviral infections in workplaces were then reported to the participants, and they were asked to report which ones they perceived as useful in order to prevent TBE, the tick-borne Lyme disease, and the mosquito-borne arboviral infection West Nile Fever (WNF). Namely (useful vs. useless): removal of standing water from the working environment; treatment of standing water with chemicals and/or biological agents; use of pesticides; use of light-colored cloths; use of full-length trousers; putting the end of trousers into the socks; self-assessment at the end of the outdoor activities.

Ethical considerations. Before giving their consent to the survey, participants were briefed that all information would be gathered anonymously and handled confidentially. Participation was voluntary, and the questionnaire was collected only from subjects who had expressed consent for study participation. As individual participants cannot be identified based on the presented material, this study caused no plausible harm or stigma to participating individuals. As the study had an anonymous, observational design, and did not include clinical data about patients, nor configured itself as a clinical trial, a preliminary evaluation by an Ethical Committee was not required, according to the Italian law (Gazzetta Ufficiale no. 76, dated 31/3/2008).

Data analysis. Continuous variables were initially tested for normal distribution (D’Agostino and Pearson omnibus normality test): where the corresponding *p* value was < 0.10, “normal” distribution was assumed as rejected, and variables were compared through Mann–Whitney or Kruskal–Wallis tests for multiple independent samples. On the other hand, variables passing the normality check (D’Agostino and Pearson *p* value ≥ 0.10) were compared using the Student’s *t* test or ANOVA, where appropriate. Categorical variables were reported as per cent values, and their distribution in respect of the outcome variable of promoting TBE vaccine was initially analyzed through chi-squared test. The paired proportions of preventive measures for TBE vs. Lyme disease, and TBE vs. the WNF were also tested through the McNemar test, assuming as the null hypothesis that no differences were reported.

All categorical variables that at univariate analysis were significantly associated with a positive attitude towards TBE vaccine (i.e., *p* < 0.05) were included in a stepwise binary logistic regression analysis model in order to calculate adjusted odds ratios (aOR) and their respective 95% confidence intervals (95%CI). All statistical analyses were performed by means of IBM SPSS Statistics 24.0 for Macintosh (IBM Corp. Armonk, NY, USA).

## 3. Results

### 3.1. Descriptive Analysis

As shown in Table 1, a total of 229 occupational physician (11.3% of the eligible population) participated to the inquiry. The majority of respondents were aged 40 years or more (66.9%); 52.0% were males, and 48.0% females. Overall, 41.9% of participants came from Northern regions (i.e., Valle d’Aosta, Piemonte, Liguria, Lombardia, Veneto, Trentino-Südtirol, Friuli-Venezia-Giulia, Emilia-Romagna), 40.6% came from Central Italy (i.e., Toscana, Umbria, Marche, Abruzzo, Lazio), and residual 17.5% from Southern regions (i.e., Campania, Molise, Puglia, Basilicata, Calabria) and major islands of Sicilia and Sardinia. Of all respondents, 44.5% resided in Italian regions characterized by incident cases of TBE, and 10.9% had any previous interaction with at least one patient affected by TBE.

### 3.2. Assessment of Knowledge about TBE

After normalization, the mean GKS was generally low (58.4% ± 12.0; actual range 30.0%–80.0%; median 60.0%). Internal consistency coefficient amounted to Cronbach’s alpha = 0.707 (Table 2).

Interestingly, only 16.6% of respondents were aware that the Italian region where they live/work is characterized by incident TBE cases, while around a quarter of participants (27.9%) acknowledged TBE as a vaccine preventable disease, and only 27.1% understood that TBE vaccine is not active against Lyme disease. Moreover, participants were affected by uncertainties about transmission of arboviruses, including the very same TBE (i.e., only 55.5% reporting tick bite as instrumental to human infection), but also dengue (only 7.0% clearly stated that dengue virus cannot be transmitted by tick bite) and Crimean-Congo hemorrhagic fever (14.4% correctly reporting tick as the infection’s primary vector). Significant uncertainties involved also the immediate managing of tick bite, as 25.3% reported that oils/lotions may improve the removal of tick head.

### 3.3. Assessment of Attitudes and Practices

A total of 44 respondents acknowledged that they usually recommend TBE vaccination in agricultural and forestry workers, i.e., 19.2% of total sample, with only 68.8% of respondents aware that the disorder may be prevented by means of vaccination.

**Figure 1:**
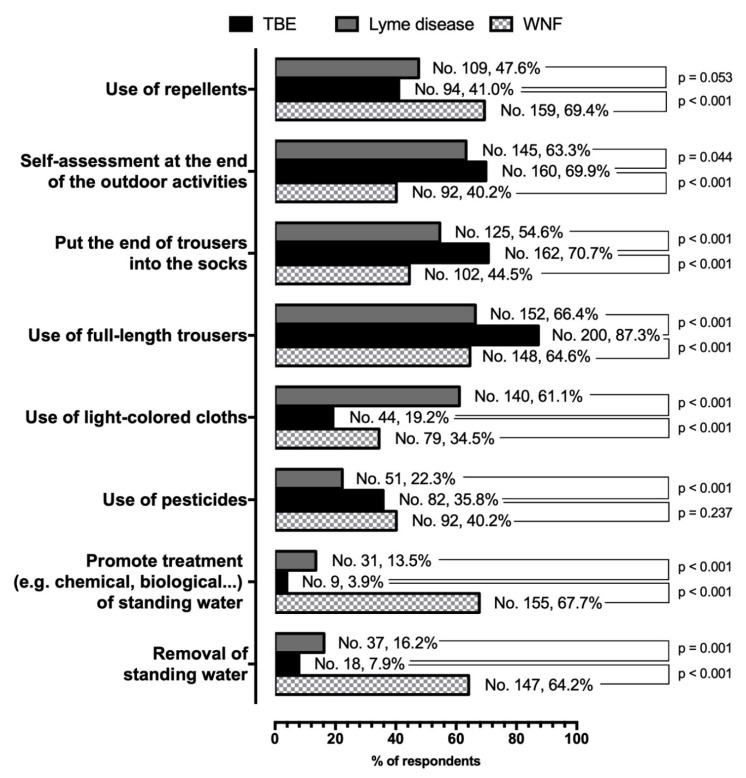
Prevalence of protective practices for TBE, Lyme disease, and West Nile Fever (WNF) reportedly recommended by 229 Occupational Physicians participating in the survey, compared by means of McNemar test for paired proportions.

Focusing on the reported preventive measures (Figure 1), TBE was associated with a distinctive pattern, even when compared to a similarly tick-borne disorder such as Lyme disease. Even though very few respondents addressed preventive treatment of standing waters, more properly reported for preventing a mosquito-borne infection such as WNF (7.9% vs. 64.2% for the removal of standing water, and 3.9% vs. 67.7% for chemical/biological treatment; both comparisons *p* < 0.001), such interventions were associated with an effective prevention of Lyme disease (16.2% for removal of standing water, and 13.5% for any other treatment, *p* = 0.001 and *p* < 0.001 compared to TBE). A significant share of respondents largely overlooked otherwise effective interventions against tick bites, such as the use of repellents (41.0% for TBE and 47.6% for Lyme disease), and the use of appropriate pesticides (35.8% and 22.3%, for TBE and Lyme disease, respectively). Both repellents and pesticides were more commonly reported against WNF (69.4%, and 40.2%); even though only for the use of repellents, the difference was statistically significant. The highly effective behavioral adaptation of promoting self-assessment at the end of outdoor activities was reported by 69.9% of respondents as effective against TBE, by 63.3% of them against Lyme disease (*p* = 0.044), and by 40.2% against WNF (*p* < 0.001). Still, we identified a mixed understanding of other behavioral adaptations, as a huge proportion of respondents advocated for TBE only countermeasures effective also against Lyme disease, such as wearing full-length trousers (87.3% vs. 66.4% for Lyme disease), and putting the end of trousers into the socks (70.7% vs. 54.6%). Less than one fifth of participants apparently promoted the use of light-colored cloths for TBE prevention (19.2%), a share significantly lower than those reported for Lyme disease (61.1%, *p* < 0.001) and even WNF (34.5%, *p* < 0.001).

### 3.4. Assessment of the Risk Perception

Overall, 16.6% of respondents acknowledged TBE severity as significant/highly significant, while 33.6% of them similarly reported its frequency as significant/highly significant. The large majority of respondents, then, did not characterize TBE as a disease of significant severity and occurrence in daily practice. A correspondent RPS equals to 20.7% ± 12.8 (range 4.0 to 60.0%) was calculated, stressing a diffuse underestimation of TBE on the workplaces.

### 3.5. Univariate Analysis

Interestingly, RPS and GKS were well correlated (*r* = 0.304, *p* < 0.001): a better knowledge status (i.e., fewer misconceptions and/or less personal attitudes guiding the vaccine decisions) was associated with a greater risk perception for TBE infection. Univariate analysis showed that promotion of TBE vaccine among agricultural and forestry workers was significantly associated with being from areas characterized by incident TBE cases (65.9% vs. 39.5% in professionals from non-endemic areas, *p* = 0.003), knowing and understanding the term “arbovirus” (81.8% vs. 57.8%, *p* = 0.005), scoring a higher GKS (50.0% in subjects with GKS > median vs. 30.8% for GKS ≤ median, *p* = 0.026). On the contrary, a proactive attitude was less frequently reported among occupational physicians acknowledging TBE as a severe disease than among those underscoring its severity (2.3% vs. 20.0%, *p* = 0.009).

### 3.6. Multivariate Analysis

In regression analysis (Table 3), being from areas endemic for TBE (aOR 3.107, 95%CI 1.473 to 6.553), knowing the term “arbovirus” (aOR 3.104, 95%CI 1.295 to 7.442), and reporting a better GKS (aOR 2.386, 95%CI 1.112 to 5.120) were identified as positive predictors for a proactive attitude towards TBE vaccination. On the contrary, acknowledging TBE as a severe disease was identified a negative effector (aOR 0.068; 95%CI 0.009–0.524).

## 4. Discussion

In our survey, the large majority of respondents was apparently unaware that a relatively uncommon but severe disease such as TBE may be avoided through an effective and reliable vaccine. As a consequence, only a small share of sampled Italian occupational physicians exhibited willingness to recommend the TBE vaccine, a feature that was substantially consistent with the relatively low acceptance for TBE vaccine even among high-risk occupational groups [20]. Main factors involved in TBE vaccine promotion among high-risk groups (i.e., agricultural and forestry workers) were identified in residing in high-risk areas, having a certain understanding about arboviruses, and exhibiting a better knowledge of TBE and tick-borne diseases. Not coincidentally, study participants exhibited a diffuse misunderstanding of TBE and its preventive measures, with risk perception significantly associated with the assessed knowledge status.

Such results were not unexpected. On the one hand, the Health Belief Model points out that personal experiences and knowledge status are the logical prerequisites to raise risk perception, which in turn promotes behavioral adaptations [22,28]. On the other hand, Occupational Physicians acknowledging TBE as a severe disease were less frequently promoting TBE vaccine among high-risk groups. These results may appear as inconsistent, or even openly in opposition with the basic assumption that a better understanding of the risk associated with a certain disorder will ultimately increase the acceptance of countermeasures [22,28], but it should be stressed that with an overall annual incidence of 0.1 cases/100,000 people, TBE remains an uncommon event for the majority of professionals not working in high-risk areas [1,2,3,4,5]. Previous studies on KAP of Italian OP have shown significant knowledge gaps and misunderstandings on up-to-date vaccinology [18,20]. Moreover, international reports hint towards diffuse false beliefs on immunization practice among such healthcare professionals [21]. Interestingly, while some previous reports have inquired KAP of medical professionals on other tick-borne disorders (e.g., Lyme disease) [29,30,31], there is a significant lack of evidence on OP and their understanding of TBE [7,14,15,16,32]. Some studies that have examined acceptance of preventive measures in endemic areas found uneven or low use of risk-reducing measures, suggesting that people at risk either ignore or underestimate the health threat from TBE, being unaware of risks and protective measures, or do not understand that the adoption of protective measures is worth their time [14,15,33,34,35,36].

Still, some aspects of the diffuse misunderstandings on TBE and its prevention were particularly frustrating, for the following reasons. First, it is obvious that while TBE may be easily prevented by avoiding high-risk habitats during the peak period of tick activity [1,9,31,37], agricultural and forestry workers may be forced to perform their tasks irrespective of such place and time restrictions. Second, TBE and Lyme disease are two distinctive disorders, for their etiology, and pathological aspects, but their prevention may benefit from the very same preventive habits. Unfortunately, sampled occupational physicians seemly assumed TBE and Lyme disease as more distinctive entities, with the potential consequence of compromising the prevention of other tick-borne viral infections, such as Crimea-Congo hemorrhagic fever, but also bacterial (e.g., *Rickettsia* spp., *Anaplasma phagocytophilum*), and protozoan agents (e.g., *Babesia microti*). Third, it should be stressed that occupational physicians are quite effective in implementing acceptance and knowledge among other high-risk workers [18,20]. Addressing knowledge gaps of occupational physicians may then maximize the consent for vaccination programs and the overcome of mutual misunderstanding between public health professionals and vaccine hesitant individuals or even vaccine objectors.

The promotion of appropriate preventive measures, either behavioral or immunization, among Italian agricultural and forestry workers, is indispensable, as some hints highlight that knowledge gaps and reported misbeliefs on TBE/TBE vaccine issues may have actively contributed to the inappropriately low protective rates otherwise recalled in previous international reports [16,24,25,32,38].

First, not only overall acceptance [20], but also national vaccination rates for TBE among high-risk workers remain quite low [2,7,9,12,14], even though serological studies have pointed out that infections do occur, also in Italian regions not usually associated with TBE [7,12,13,15,16]. Interestingly, in some previous studies, the most commonly reported reason for not being vaccinated against TBE was the unawareness that a vaccine exists [20], despite a quite good interaction with healthcare providers such as the general practitioner and the occupational physician [14,31]. In other words, while our results seemingly confirm the earlier suspect that healthcare professionals (particularly occupational physicians) are not actively promoting TBE immunization in high–risk workers groups, since these professionals show a diffuse neglect of such prevention strategy, vaccination rates may be significantly improved by enriching their knowledge about TBEV and TBE vaccine [7,14,20,31].

Second, a previous report from North-Eastern Italy pointed out that a high percentage of farmers usually avoid behavioral protective and preventive measures [14,33,35]. Despite half of participants recalled tick bites while performing their work tasks [14], the systematic body checks for tick bites after field work (i.e., 2.8%) was often not performed [33,36,38,39]. Keeping in mind the increasing threat represented by TBE in some Italian Regions, but also that *Borrelia burgdorferi* actively circulates among Italian isolates of *Ixodes ricinus*, and reliable vaccines for Lyme disease still remain unavailable, an appropriate intervention of occupational physicians on such specific topic has, therefore, the potential to significantly improve health and safety of agricultural and forestry workers. Despite the fact that TBEV infection occurs in the first minutes after a tick bite, the virus begins to multiply actively in the feeding tick, increasing the infection dose, and, in turn, increasing the risk of disease. This means that the rapid removal of ticks reduces the risk not only for bacterial infections, but also for TBE [40]. For this reason, understanding and sharing appropriate methods for the removal of attached ticks may extensively reduce the overall risk for the majority of tick borne infections [34,41].

Our study shows several limitations. Firstly, it shares the implicit limits of Internet-based surveys [42,43]. Such studies are actually reliable, cost-effective, and mostly much faster than a paper-based survey. However, as participants are somehow “self-selected”, the final sample may potentially over-represent some sub-groups (e.g., subjects from younger age groups, with a greater literacy, and more accustomed to the internet access), eventually failing to represent the original population. Therefore, a significant selection bias cannot be ruled out. Again, as in conventional paper-based surveys, participating voluntarily could be due to a proactive attitude or greater knowledge about vaccinations. In the same way, the fact of not participating could be understood as a negative attitude or a lack of knowledge about vaccinations.

Again, we cannot rule out that some of the items assessed through the knowledge test may be affected by a significant social desirability bias, with participants reporting the “socially appropriated” rather than their authentic behaviors, so that our results could have ultimately overstated the share of occupational physicians having an effective understanding of TBE associated issues [14,44,45].

Moreover, our sample was of limited size, including only 229 professionals among the over 7000 occupational physicians from the national list [20]. Even though we required the participants to report their geographic origin, such factor was assessed among the possible effectors for TBE-related KAP, without any role in the sampling strategy. As Italy has been repetitively acknowledged for very heterogeneous health literacy and vaccination rates, our results should be cautiously interpreted as representative of the national level [17,18,46]. Similarly, while a certain selection is usually performed by social media managers of specific discussion groups (e.g., by registering only subjects who receive a specific invitation by the manager; answering to specific “selection” questions; etc.), we cannot rule out that some of the respondents were not actively working as occupational physicians, limitedly or even not fulfilling our initial selection criteria.

Finally, collected data were not externally validated, lacking an estimate of high-risk workers actually followed by sampled occupational physicians. More specifically, we are neither able to ascertain how often sampled professionals interact with agricultural and/or forestry workers, nor which share of their practice they actually represent. Similarly, we are unable to assess how reliable are the practices reported by respondents, that is which share of workers followed by participants actually receive vaccines and/or specific recommendations. As a consequence, we were unable to estimate the effective extent of the social desirability bias, being the actual vaccination rates for TBE potentially even lower than those self-reported by study participants.

## 5. Conclusions

In conclusion, our results suggest an extensive lack of knowledge of sampled occupational physicians on TBE vaccination. Such results are consistent with previous reports on the TBE and its preventive measures in Northern Italy, and with the limited evidence on vaccine literacy of occupational physicians. More specifically participants were seemly unaware that an effective TBE vaccine does exist, and underestimated the potential health risks associated with TBEV infection. Our results suggest that a significant share of occupational physicians actually ignores or only partially applies official recommendations on TBE vaccinology. As knowledge status appeared well correlated with risk perception, being moreover characterized as a significant predictor of a proactive attitude towards TBE among occupational physicians, it is reasonable that filling their information gaps might improve the rate of proper preventive measures among agricultural and forestry workers. As TBEV infection may be effectively countered not only by means of TBE vaccine, but also through effective behavioral practices, improving this way the prevention of all tick-borne infections, increasing vaccination literacy of occupational physicians and promoting their interaction with agricultural and forestry workers would be, therefore, instrumental and cost-effective in reducing the potential spreading of all tick-borne disorders.

## Figures and Tables

**Table 1:** Characteristics of 229 Italian occupational physicians participating in the survey (2020). Likert scale for perceived severity and perceived frequency of tick-borne encephalitis (TBE) in agricultural and forestry settings were dichotomized as “significant” and “very significant” (i.e., severe and frequently reported disease) vs. all other values (i.e., not severe and infrequently reported).

Variable	No., %	Average ± SD
**Age Group**		
*<30 years*	10, 4.4%	
*30–39 years*	66, 28.8%	
*40–49 years*	102, 44.5%	
*50–59 years*	38, 16.6%	
*≥60 years*	13, 5.7%	
**Gender**		
*Male*	119, 52.0%	
*Female*	110, 48.0%	
**Residence**		
*Northern Italy*	96, 41.9%	
*Central Italy*	93, 40.6%	
*Southern Italy*	40, 17.5%	
**Residence in Italian Region Endemic for TBE**	102, 44.5%	
**Knowledge of the Term “arbovirus”**	143, 62.4%	
**Any Previous Interaction with TBE case(s) in the Practice**	25, 10.9%	
**TBE Immunization Recommended in High-Risk Occupational Groups**	44, 19.2%	
**General Knowledge Score**		58.4% ± 12.0 (median 60.0%)
**General Knowledge Score > Median** (i.e., 60.0%)	79, 34.5%	
**TBE Acknowledged as a Severe Disease**	38, 16.6%	
**TBE Acknowledged as a Frequently Reported Disease**	77, 33.6%	
**Risk Perception Score**		20.7% ± 12.8 (median 16.0%)

Notes: TBE = Tick Borne Encephalitis; Northern Italy = Italian regions of Valle d’Aosta, Piemonte, Liguria, Lombardia, Veneto, Trentino-Südtirol, Friuli-Venezia-Giulia, Emilia-Romagna; Central Italy = Italian regions of Toscana, Umbria, Marche, Abruzzo, Lazio; Southern Italy = Italian regions of Campania, Molise, Puglia, Basilicata, Calabria, Sicilia, Sardinia.

**Table 2:** Knowledge test: response distribution of presented items proposed to the 229 Occupational Physicians participating in the survey and contributing to the assessment of General Knowledge Score (GKS) (Cronbach’s Alpha = 0.707).

Statement	Correct Answer	No., %
1. The subsequent disorders are transmitted by tick bite		
*TBE*	TRUE	127, 55.5%
*Crimea-Congo Hemorrhagic Fever*	TRUE	33, 14.4%
*Lyme Disease*	TRUE	173, 75.5%
*Yellow Fever*	FALSE	219, 96.5%
*Dengue*	FALSE	16, 7.0%
*West Nile Fever*	FALSE	223, 97.4%
2. The subsequent disorders are preventable through commercially available vaccination(s)		
*TBE*	TRUE	64, 27.9%
*Crimea-Congo Hemorrhagic Fever*	FALSE	198, 86.5%
*Lyme Disease*	FALSE	205, 89.5%
*Yellow Fever*	TRUE	130, 56.8%
*Dengue*	FALSE	175, 76.4%
*West Nile Fever*	FALSE	183, 79.9%
3. In the Italian Region where he/she lives/works TBE is endemic	*	38, 16.6%
4. In cases of tick bites, the head should be removed as soon as possible	TRUE	184, 80.3%
5. Tick head should be removed by means of specifically designed tweezers	TRUE	157, 68.6%
6. Tick head removal may be improved by means of oils/lotions	FALSE	58, 25.3%
7. TBE vaccine is effective also against Lyme disease	FALSE	62, 27.1%
8. Arboviral infections in at-risk professionals are compensated as occupational injuries	TRUE	123, 53.7%
9. TBE is characterized by inter-human spreading	FALSE	174, 76.0%
10. Latency for Lyme’s disease may be of weeks up to some months	TRUE	135, 59.0%

Note = * depending of the region of residence.

**Table 3:** Analysis of factors associated with promoting tick-borne encephalitis (TBE) vaccine in 229 Italian Occupational Physicians (2020).

Variable	TBE Immunization Recommended in High-Risk Workers		Chi Squared Test *p* Value	aOR (95% CI)
	Yes (No./44, %)	No (No./185, %)		
**Age < 40 Years**	13, 29.5%	63, 34.1%	0.695	-
**Male Gender**	18, 40.9%	101, 54.6%	0.143	-
**Residence in Northern Italy vs. Other Regions**	24, 54.5%	72, 38.0%	0.086	-
**Residence in Italian Region Endemic for TBE**	29, 65.9%	73, 39.5%	0.003	3.107 (1.473; 6.553)
**Knowledge of the Term “arbovirus”**	36, 81.8%	107, 57.8%	0.005	3.104 (1.295; 7.442)
**Any Previous Interaction with TBE case(s) in the Practice**	5, 11.4%	20, 10.8%	1.000	-
**General Knowledge Score > Median**	22, 50.0%	57, 30.8%	0.026	2.386 (1.112; 5.120)
**TBE Acknowledged as a Severe Disease**	1, 2.3%	37, 20.0%	0.009	0.068 (0.009; 0.524)
**TBE Acknowledged as a Frequently Reported Disease**	11, 25.0%	66, 35.7%	0.242	-

Notes: aOR = adjusted Odds Ratio (i.e., Odds Ratio calculated through binary logistic regression).
